# The clinical and genetic spectrum of primary familial brain calcification

**DOI:** 10.1007/s00415-023-11650-0

**Published:** 2023-03-02

**Authors:** Miryam Carecchio, Michele Mainardi, Giulia Bonato

**Affiliations:** Department of Neuroscience, University of Padua, Via Niccolò Giustiniani, 5, 35128 Padua, Italy

**Keywords:** PFBC, Brain calcification, Fahr, Genetics, Phenotype

## Abstract

Primary familial brain calcification (PFBC), formerly known as Fahr’s disease, is a rare neurodegenerative disease characterized by bilateral progressive calcification of the microvessels of the basal ganglia and other cerebral and cerebellar structures. PFBC is thought to be due to an altered function of the Neurovascular Unit (NVU), where abnormal calcium-phosphorus metabolism, functional and microanatomical alterations of pericytes and mitochondrial alterations cause a dysfunction of the blood–brain barrier (BBB) and the generation of an osteogenic environment with surrounding astrocyte activation and progressive neurodegeneration. Seven causative genes have been discovered so far, of which four with dominant (*SLC20A2*, *PDGFB*, *PDGFRB*, *XPR1*) and three with recessive inheritance (*MYORG*, *JAM2*, *CMPK2*). Clinical presentation ranges from asymptomatic subjects to movement disorders, cognitive decline and psychiatric disturbances alone or in various combinations. Radiological patterns of calcium deposition are similar in all known genetic forms, but central pontine calcification and cerebellar atrophy are highly suggestive of *MYORG* mutations and extensive cortical calcification has been associated with *JAM2* mutations. Currently, no disease-modifying drugs or calcium-chelating agents are available and only symptomatic treatments can be offered.

## Introduction

Primary familial brain calcification (PFBC) is a rare neurodegenerative disease characterized by bilateral calcium deposition in the basal ganglia, with possible additional involvement of the cerebellar dentate nuclei, thalami, subcortical white matter, cerebral and cerebellar cortex. Mean age of clinical onset is around 40–50 years and estimated prevalence is < 1/1.000 [1].

PFBC was formerly known as Fahr’s disease, after the name of the German neuropathologist Karl T. Fahr who described in 1930 an elderly patient with dementia, motor problems and post-mortem detection of extensive calcium deposition in basal ganglia, striatum and white matter probably due to calcium-phosphorus abnormalities [2]. However, basal ganglia calcifications in a man with tremor, rigidity and lower legs weakness had already been reported on autopsy in 1850 by Delacour [3]. A possible genetic aetiology was postulated in 1977, when Boller reported a family with nine affected subjects with an autosomal dominant pattern of inheritance [4]. Several different terms have been used to describe this nosological entity, including Idiopathic Basal Ganglia Calcifications (IBCG) [5] and bilateral striato-pallido-dentate calcinosis (BSPCD) [6]. The term PFBC, which has replaced the term “Fahr’s disease/syndrome”, was introduced in 2013 and applies to cases of calcium deposition in the central nervous system with a genetic aetiology [7] and is now used in view of the genetic advances of the last ten years in this field.

## Neuropathology and genetics

The main neuropathological feature of PFBC is the presence of calcified nodules along the capillaries at the neurovascular unit, the microanatomic structure constituting the blood–brain barrier (BBB). At this level, endothelial cells are surrounded by basement membrane, pericytes and astrocyte end-feet; microglia are in contact with the NVU by means of cytoplasmatic processes. An altered function of pericytes has been demonstrated in animal models of PFBC and is now thought to have a central role in its pathogenesis [8]. Pericytes are capillary wall cells embedded in the endothelial basement membrane that express certain markers, including PDGF-Rb, encoded by one of the genes responsible for autosomal dominant cases of PFBC. These cells are required for the formation of vessels during embryonic development, and, in the adult vasculature, they have several roles in the brain, including blood flow modulation and regulation of the BBB [9–11].

A dysfunction of the NVU triggered by local formation of calcified nodules is thought to be the main mechanism eventually leading to neurodegeneration in subjects carrying mutations in PFBC-associated genes. In a murine model of PFBC due to *PDGFB* mutations, Zarb et al. [12] characterized the cellular environment surrounding calcifications, demonstrating that cells around vessel-associated calcifications express markers for osteoblasts, osteoclasts and osteocytes, leading to the presence of an osteogenic environment and a progressive ossification process at the NVU. Calcifications were also observed to cause oxidative stress in astrocytes and trigger the expression of neurotoxic astrocyte markers, thus leading to progressive neurodegeneration, as reflected by the progressive clinical nature of this neurological condition. It is thought that mutations in PFBC-related genes, through different pathways, lead to a functional disruption of the neurovascular unit, pericyte deficiency and altered properties of the endothelial cells. Interestingly, skin biopsies from patients carrying mutations in different PFBC genes have shown the presence of a microangiopathy with microcalcifications in the basal lamina, within and around the pericytes as observed on electron microscopy [13, 14].

PFBC cases can be sporadic or inherited with an autosomal dominant (AD) or recessive (AR) pattern. Dominantly inherited PFBC is associated with mutations in four genes: solute carrier 20 member 2 (*SLC20A2*) [15], xenotropic and polytropic retrovirus receptor 1 (*XPR1*) [16], platelet-derived growth factor B (*PDGFB*) [17] and platelet-derived growth factor receptor B (*PDGFRB*) [18].

Recessively inherited PFBC is caused by three genes: Myogenesis Regulating Glycosidase protein (*MYORG*) [19], Junctional Adhesion Molecule 2 *(JAM2)* [20], and a recently published new gene, cytidine monophosphate (UMP-CMP) kinase 2 (*CMPK2*) [21].

The *SLC20A2* gene, located on chromosome 8 (8p11.21), codes for the type III Na-dependent inorganic phosphate (Pi) transporter 2 (PiT2), which is strongly expressed in neurons, but also in astrocytes and endothelial cells in parts of the brain commonly affected by PFBC, such as basal ganglia. PiT2 is a transmembrane Na + /Pi cotransporter that contributes to the maintenance of Pi homeostasis, which is essential for adenosine triphosphate synthesis. Mutations in this gene result in impaired uptake of inorganic phosphate, leading to its extracellular accumulation and precipitation of calcium phosphate in the vascular extracellular matrix [15].

*XPR1* is located on chromosome 1 (1q25) and encodes a trans-membrane protein mediating the export of inorganic phosphate from the intracellular toward the vascular extracellular matrix [22, 23].

The *PDGFB* and *PDGFRB* signalling axis is essential during embryonic development and early post-natal life in regulating pericyte formation and recruitment along newly forming vessels. Animal models lacking PDGFB/PDGFRB expression show reduced pericyte coverage of blood vessels, suggesting that the integrity of this signalling pathway is required for a normal anatomical and functional development of the neurovascular unit [8]. During adult life, pericytes exert several functions in the regulation of the BBB at the NVU, including blood flow regulation, possible formation of endothelial junctions, and astrocytic end-foot polarization [11, 24].

The *MYORG* gene, also known as *KIAA1161* or *NET37*, maps to chromosome 9 (9p13.13) and it is expressed in astrocytes and in various cell lines. It has been shown to be distributed to the endoplasmic reticulum (ER) and nuclear envelope. The *MYORG* biochemical function has recently been elucidated: it is an α-galactosidase with an elevated substrate specificity for human glycans containing the disaccharide Gal-α1,4-Glc [25]. Its role in the pathogenesis of PFBC may be due to an altered quality control process on the folding or maturation of one or more of the protein products of genes linked to PFBC including *SLC20A2*, *PDGFB*, *PDGFRB*, and *XPR1* [15–18], which are, in fact, all glycoproteins.

*JAM2* encodes junctional adhesion molecule 2, which is highly expressed in neurovascular unit- related cell types (endothelial cells and astrocytes) and predominantly localizes on the plasma membrane. Junctional adhesion molecules play an important role in the regulation of cell polarity, endothelium permeability, and leukocyte migration and the blood–brain-barrier (BBB) function. Mutations in *JAM2* may result in impaired cell-to-cell adhesion function and altered integrity of the NVU ultimately leads to BBB dysfunction and brain calcification at this level [20, 26]. Interestingly, biallelic mutations in other genes belonging to the junctional adhesion molecule family (*JAM3* and *OCLN*) have been linked to complex neurological diseases characterized by brain calcification, suggesting that an altered function of molecules belonging to this family leads to a final common pathway causing cerebral calcification in humans [27, 28].

*CMPK2* is a recessively inherited gene very recently discovered in two unrelated Chinese families with three affected subjects [21]. CMPK2 is highly expressed in neurons and endothelial vascular cells. Its reduced expression in a mutant animal knock-out mouse model has been shown to lead to a reduced number of mitochondrial DNA copies, down-regulated mitochondrial proteins, reduced ATP production, and elevated intracellular inorganic phosphate (Pi) level, causing progressive intracranial calcification.

## Clinical and radiological features

PFBC can present with movement disorders, cognitive decline, psychiatric manifestations and cerebellar signs, but patients can also be asymptomatic despite extensive cerebral calcification [29]. In fact, pathogenic variants in PFBC-related genes are 100% radiologically penetrant but they exhibit reduced clinical penetrance, with some differences related to the specific underlying mutated gene.

The clinical phenotype has been reviewed in a series of 516 genetically confirmed PFBC patients, of which 67.6% were clinically affected [30]. Data from this review indicated a clinical penetrance of > 85% in *MYORG*, *PDGFB* and *JAM2* patients, whereas *SLC20A2* and *XPR1* patients had a penetrance of 60% and 66%, respectively. From available data, overall median age at onset was 43 years; this was significantly lower in *PDGFB* mutation carriers as compared to other genes.

Motor manifestations were reported in about one third of clinically affected patients and in 42% of cases they were associated with non-motor symptoms/signs (anxiety, psychosis, cognitive decline, headache).

Parkinsonism is the main motor phenotype in PFBC, but chorea and dystonia were reported in 20% of symptomatic cases in a series of 137 patients [31]. Seizures, cerebellar and pyramidal signs have also been frequently described. Dysarthria (even isolated) is almost universally present in symptomatic *MYORG*-mutation carriers [32, 33] that tend to display a phenotype dominated by progressive cerebellar signs with ataxia and cognitive decline. MYORG patients can also show a phenotype similar to progressive supranuclear palsy (PSP) with vertical gaze palsy, progressive cognitive decline and early falls in the context of akinetic-rigid parkinsonism [34]. Neuropsychiatric disturbances include various degree of cognitive impairment, mood disorders and other psychiatric disorders.

Clinical manifestations associated with mutations in *CMPK2* have been recently described in three affected subjects [21]. Two out of three patients presented with a combination of ataxia, dysarthria, motor dysfunction and severe cognitive decline starting in the third-to-fourth decade and progressing over a few years to an advanced-stage neurological deterioration.

**Fig. 1:**
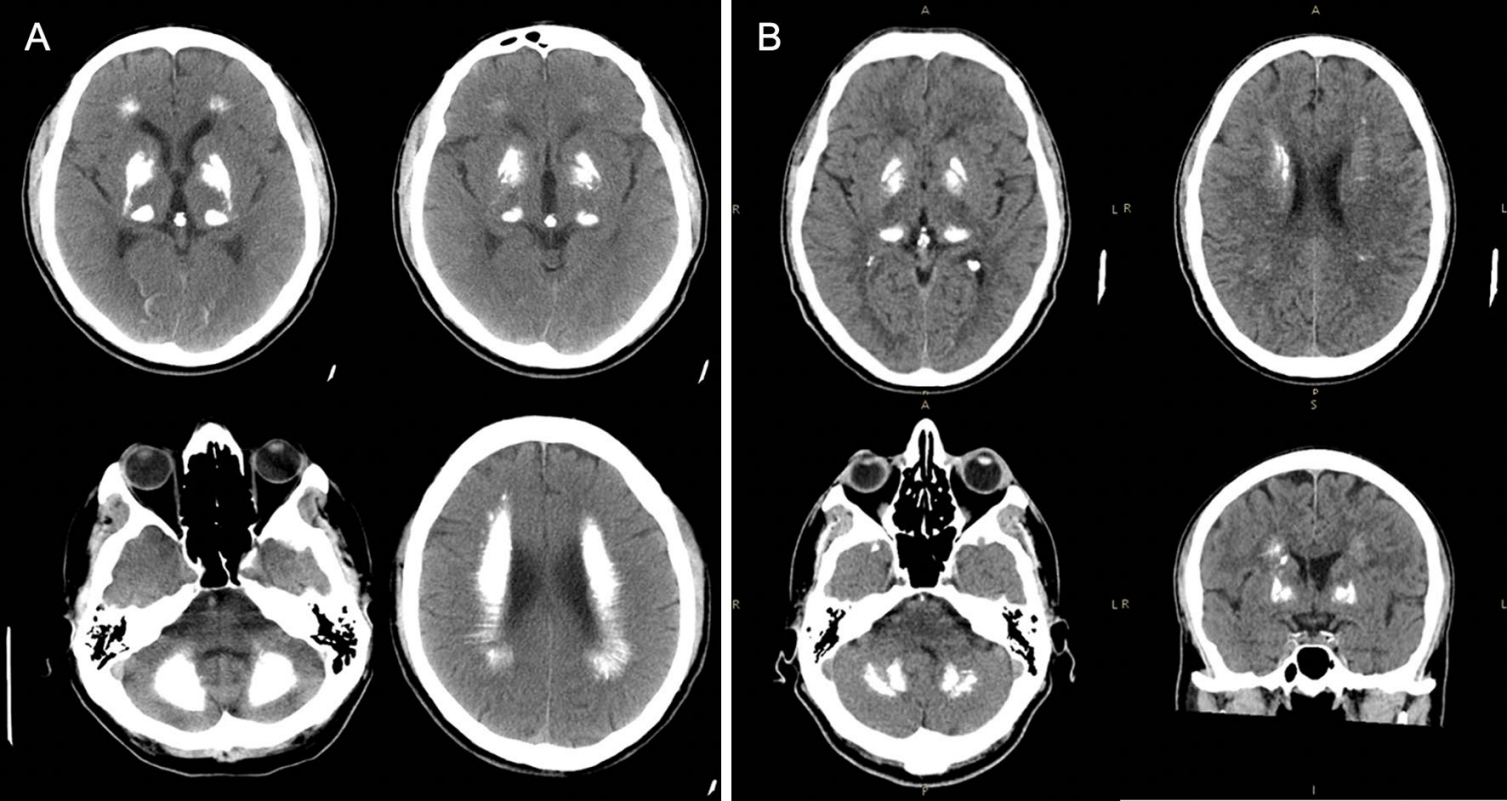
Brain CT scan of: **A** a patient carrying a pathogenic *SLC20A2* mutation with extensive bilateral calcifications of the putamen, globus pallidus, thalami, cerebellar dentate nuclei, subcortical white matter and occipital cortex; **B** a patient carrying a pathogenic mutation of *PDGFRB* with calcifications of globus pallidum interna, thalami, cerebellar dentate nuclei and with mild involvement of the subcortical white matter

As for radiological features, bilateral basal ganglia calcifications are detectable in all PFBC patients as hyperdense lesions, with varying degrees of involvement of additional brain areas, especially the cerebellar dentate nuclei, thalami, subcortical white matter and cerebral cortex (Fig. 1). CT scans can discriminate calcium deposition better than brain MRI, on which different paramagnetic materials (such as iron and calcium) indistinctly appear hypointense on SWI sequences. The Total Calcification Score (TCS), a semi-quantitative visual scale, has been proposed which strives to quantify the extent of brain calcification [29].

Mutations in different PFBC-related genes can be associated with specific radiological features. Biallelic mutations in *MYORG* and *JAM2* have been associated with a more severe and extensive calcium deposition pattern compared to dominant genes, with prominent cortical and cerebellar involvement. Central pontine calcifications are highly suggestive of *MYORG* mutations that also cause a significant degree of cerebellar atrophy and seem to generate a more severe phenotype [32, 34]. *JAM2* mutation carriers exhibit a higher TCS as compared to *MYORG* patients, with severe and confluent cortical calcification involving the occipital, temporal, frontal and parietal cortices bilaterally [20].

The extension and number of calcified cerebral areas positively correlates with the patients’ age [29, 30], but this does not always translate into a worse clinical presentation.

As for *CMPK2* mutation carriers, available data suggests there is an elevated TCS (> 50) in symptomatic subjects with calcifications extending to the subcortical white matter bilaterally [21].

Interestingly, healthy carriers of heterozygous *MYORG* mutations have been shown to have small-sized basal ganglia calcifications mainly involving the GPi that could be overlooked in clinical practice as paraphysiological, age-related brain calcifications [32, 34]. Also, some heterozygous *CMPK2* carriers from two recently published Chinese families exhibit small, punctate calcification of the GPi on brain CT scan [21]. Such a radiological finding has also been reported in two single *JAM2* mutation carriers who were clinically symptomatic although the genetic analysis failed to identify a second pathogenic variant [35]. Taken together, this observation suggests that monoallelic mutations in recessive PFBC-related genes may be radiologically relevant and exhibit a semi-dominant inheritance pattern with reduced clinical penetrance, but a systematic radiological assessment of heterozygous mutation carriers is still lacking in the available literature.

PET-FDG studies in PFBC patients have shown basal ganglia hypo-metabolism with some cortical involvement especially in patients with cognitive decline [36]. DAT-Scan alterations indicating nigro-striatal degeneration have not been systematically assessed in PFBC patients and available data from small cohorts or case reports have inconsistently shown an altered tracer uptake [37, 38] in patients with parkinsonism.

## Diagnosis

The diagnosis of PFBC is based on the demonstration of bilateral basal ganglia calcifications on brain imaging (CT scan) and the exclusion of secondary causes of calcium deposition in the brain. Abnormal clinical findings on examination, as well as a positive family history of brain calcification, neurological or psychiatric disturbances can be absent; hence, diagnosis can be formulated on radiological grounds only after ruling out secondary causes. These include persistent hypocalcaemia due to calcium metabolism alterations, infectious and mitochondrial diseases, as well as other rare neurodegenerative conditions. Differential diagnosis is based on the patients’ age, clinical history, examination and laboratory findings. Laboratory screening should include a full calcium metabolism assessment (parathyroid hormone, vitamin D, calcium and phosphate levels), lactic acid and CPK (possibly increased in mitochondrial diseases) along with brain CT scan that is the gold standard radiological method to visualize cerebral calcification [39].

Age-related basal ganglia calcification, especially of the internal globus pallidus (GPi) are considered paraphysiological and can be found in up to 15–20% of the elderly population with no significant clinical correlates of basal ganglia dysfunction [40].

The main differential diagnosis of basal ganglia calcification includes:

1. **Disorders of calcium metabolism**. Low levels of parathyroid hormone (PTH), the main hormone involved in calcium-phosphate metabolism, is the main differential diagnosis in adult patients. Persistent hypoparathyroidism or altered response to PTH (pseudohypoparathyroidism) leads to low blood calcium levels and high phosphate, thus promoting calcium phosphate crystals deposition. Hypoparathyroidism can be idiopathic or secondary to accidental parathyroid glands’ excision during thyroid surgery [41]. The main clinical manifestations include paraesthesia, cramps, carpo-pedal spasms, seizures and arrhythmia. Up to 74% of patients with idiopathic hypoparathyroidism develop brain calcifications on CT scans that are indistinguishable from PFBC and can be associated with the same symptoms [42]. Post-surgical hypoparathyroidism is reported in up to 1.5% of patients undergoing total or sub-total thyroidectomy [43].
  Pseudo-hypoparathyroidism is a genetic disorder caused by mutations in the *GNAS* and *STX16* genes, characterized by peripheral PTH resistance (hypocalcaemia with normal or high plasmatic levels of PTH), causing intellectual disability and Albright osteodystrophy (short stature, obesity, systemic tissues calcification, hypogonadism) [44].
2. **Infectious diseases**. Toxoplasmosis, rubella, cytomegalovirus and herpes simplex virus (TORCH complex) can cause brain calcifications, as well as cysticercosis and neurobrucellosis. HIV-related calcifications affect vessels of multiple organs including the brain; basal ganglia calcifications are rare in adults but can be found in up to 30% of the paediatric cases [45].
3. **Paediatric causes**. Genetically determined, congenital disorders associated with basal ganglia calcification include Cockayne and Aicardi-Goutières syndromes. Cockayne Syndrome is a genetically heterogeneous disorder partially overlapping with Xeroderma Pigmentosum caused by biallelic mutations in genes regulating DNA repair (*ERCC6*, *ERCC8*). It is classified among the childhood-onset leukodystrophies and is characterized by diffuse white matter hypomyelination on brain MRI along with putaminal, cerebellar and cortical calcification [46]. Patients present with a variety of manifestations including developmental delay, intellectual disability, peripheral neuropathy, sensorineural hearing loss, ataxia and spasticity. Non neurological manifestations include retinitis pigmentosa, skin photosensitivity and dysmorphic features.
  Aicardi-Goutières syndrome (AGS) is a paediatric recessive encephalopathy classified among type I interferonopathies, a genetically heterogeneous group of autoinflammatory disorders characterized by cerebrospinal fluid chronic lymphocytosis and raised levels of interferon-alpha. The main neuroradiological features include basal ganglia calcification, leukoencephalopathy and cerebral atrophy. Clinical manifestations include developmental delay, neuromuscular problems, epilepsy, pyramidal signs, fever and vasculo-cutaneous alterations in the limbs [47].
4. **Mitochondrial disorders**. MELAS (mitochondrial encephalopathy, lactic acidosis, and stroke-like episodes), MERRF (myoclonic epilepsy with ragged red fibres) and Kearns-Sayre syndrome are characterized by high levels of serum lactic acid and calcification of basal ganglia, especially the globus pallidus interna that can occur in up to 13% of cases [48]. MELAS is most often caused by a 3243A-G transition in the *MTTL1* gene; MERFF is caused in up to 90% of cases by an A-G mutation at nucleotide 8344 of the *MTTK* gene. The presence of other typical symptoms and multisystemic features usually point to a mitochondrial aetiology [49].
5. **Metal deposition**. Other rare genetic syndromes with metal depositions in the basal ganglia, such as iron and manganese, should also be considered in the differential diagnosis since they can have similar features on brain MRI, even though the age of onset is usually earlier than PFBC and CT scans can help distinguish the nature of the deposition. Among these, Neurodegeneration with Brain Iron Accumulation syndromes (NBIA), PKAN (pantothenate-kinase associated neurodegeneration, due to biallelic *PANK2* mutations) can be characterized by punctate calcium deposition in the basal ganglia over the underlying iron accumulation [50]. Some cases of calcifications have been reported also in BPAN (Beta-Propeller Associated Neurodegeneration, due to mutations in *WDR45*) in patents with dystonia and neuropsychiatric features [51].
6. **Other adult-onset neurodegenerative disorders**. Additional, rare neurodegenerative diseases characterized by intracranial calcification include SCA20 (isolated calcium deposition in cerebellar dentate nuclei without basal ganglia involvement), neuroferritinopathy due to *NFT* mutations, polycystic lipomembranous osteodysplasia with sclerosing leukoencephalopathy (Nasu Hakola disease) and diffuse neurofibrillary tangles with calcifications (Kosaka-Shibayama disease).

## Clinical management

Unlike other neurodegenerative diseases, PFBC can be radiologically manifest with severe calcium deposition in various cerebral areas but with no evidence of neurological dysfunction. This mismatch can be problematic for genetic counselling, since the disease can be diagnosed based on calcification on brain CT scan in totally asymptomatic subjects and no prognostic factors to predict the future development of neurological and psychiatric symptoms are currently known. Nevertheless, genetic analyses and counselling allows to predict the risk of transmission of known mutations to offspring, thus estimating the risk of finding brain calcifications in relatives of affected patients and offering them an early neurological follow up.

Radiological follow-up of PFBC patients with brain CT or MRI scan has limited value in clinical practice, since no therapeutical decision is made based on radiological findings and it is not possible to establish disease prognosis even in cases with demonstratable extension of calcification on brain imaging over time.

To date, no specific therapies are available to chelate calcium in the brain, and no disease modifying therapies or prevention strategies are known.

Nimodipine, a calcium channel blocker in the CNS, has been unsuccessfully tried to attenuate PFBC symptoms [52]. Bisphosphonates, commonly used for the treatment of osteoporosis, have been reported to improve symptoms in single cases, without a demonstrated reduction in calcium deposition on CT scans [53]. In a series of seven patients, Oliveira et al. observed stabilization of disease progression after administering alendronate without significant side effects, but no changes on brain calcium deposition were observed and no control group was used for comparison [54].

At present, treatment of PFBC-related symptoms remains symptomatic. Antipsychotics and antidepressants are used for neuropsychiatric disturbances [53]. In patients with parkinsonism, L-Dopa has been used with variable response. Cases of significant improvement have been reported in patients with altered DaT-Scan imaging [55–57], whereas no clinical benefit has been reported in patients with cerebellar signs, atypical features and gait disturbances [58, 59].

## Conclusions

PFBC is a clinically and genetically heterogeneous disorder characterized by progressive cerebral and cerebellar calcification that can manifest with a wide range of symptoms or be asymptomatic. Radiological and clinical features often do not match up even in elderly subjects with long-standing evidence of cerebral calcification on imaging. The genetic bases of this disease started being elucidated in 2012, and seven causative genes are now known, although a proportion of cases still lack a genetic diagnosis, due to the likely existence of still unknown genes.

Whole exome sequencing (WES) and customized NGS panels are useful diagnostic tools in identifying mutations in genes related to PFBC or other rare conditions featuring intracranial calcification, thus allowing for a precise molecular diagnosis and genetic counselling to affected subjects. Having a hallmark of the disease even in asymptomatic subjects, namely radiological evidence of basal ganglia calcification can help to determine the pathogenicity of variants of unknown significance (VUS) highlighted by genetic analysis. For this reason, genetic testing in patients’ relatives should always be accompanied by brain CT scan.

The development of disease-specific drugs to target altered pathways in PFBC is a major challenge in this field that partially overlaps with several other genetic human diseases characterized by abnormal calcium deposition in human tissues.
